# Post-Concussive Vestibular Dysfunction Is Related to Injury to the Inferior Vestibular Nerve

**DOI:** 10.1089/neu.2021.0447

**Published:** 2022-06-03

**Authors:** Anna Gard, Ali Al-Husseini, Evgenios N. Kornaropoulos, Alessandro De Maio, Yelverton Tegner, Isabella Björkman-Burtscher, Karin Markenroth Bloch, Markus Nilsson, Måns Magnusson, Niklas Marklund

**Affiliations:** ^1^ Department of Clinical Sciences Lund, Lund University, Neurosurgery, Skåne University Hospital, Lund, Sweden.; ^2^ Department of Clinical Sciences Lund, Diagnostic Radiology, Lund University, Skåne University Hospital, Lund, Sweden.; ^3^ Department of Radiological, Oncological and Pathological Sciences. Policlinico Umberto I, Sapienza University of Rome, Rome, Italy.; ^4^ Department of Health Sciences, Luleå University of Technology, Luleå, Sweden.; ^5^ Department of Radiology, Institute of Clinical Sciences, Sahlgrenska Academy, University of Gothenburg, Gothenburg, Sweden.; ^6^ Lund University Bioimaging Center, Lund University, Lund, Sweden.; ^7^ Department of Clinical Sciences Lund, Otorhinolaryngology, Lund University, Skåne University Hospital, Lund, Sweden.

**Keywords:** concussion, inferior vestibular nerve, persisting post-concussion symptoms, 7T MRI, sports-related concussion, vestibular dysfunction

## Abstract

Symptoms of vestibular dysfunction such as dizziness and vertigo are common after sports-related concussions (SRC) and associated with a worse outcome and a prolonged recovery. Vestibular dysfunction after SRC can be because of an impairment of the peripheral or central neural parts of the vestibular system. The aim of the present study was to establish the cause of vestibular impairment in athletes with SRC who have persisting post-concussive symptoms (PPCS). We recruited 42 participants—21 athletes with previous SRCs and PPCS ≥6 months and 21 healthy athletic age- and sex-matched controls—who underwent symptom rating, a detailed test battery of vestibular function and 7T magnetic resonance imaging with diffusion tensor imaging (DTI) and diffusion kurtosis imaging (DKI) of cerebellar white matter tracts, and T1-weighted imaging for cerebellar volumetrics. Vestibular dysfunction was observed in 13 SRC athletes and three controls (*p* = 0.001). Athletes with vestibular dysfunction reported more pronounced symptoms on the Dizziness Handicap Inventory (DHI; *p* < 0.001) and the Hospital Anxiety and Depression Scale (HADS; *p* < 0.001). No significant differences in DTI metrics were found, while in DKI two metrics were observed in the superior and/or inferior cerebellar tracts. Cerebellar gray and white matter volumes were similar in athletes with SRC and controls. Compared with controls, pathological video head impulse test results (vHIT; *p* < 0.001) and cervical vestibular evoked myogenic potentials (cVEMP; *p* = 0.002) were observed in athletes with SRC, indicating peripheral vestibular dysfunction and specifically suggesting injury to the inferior vestibular nerve. In athletes with persisting symptoms after SRC, vestibular dysfunction is associated with injury to the inferior vestibular nerve.

## Introduction

Contact sport athletes are at high risk of sustaining head impacts that may lead to a sports-related concussion (SRC).^1^ The incidence of SRC has increased during the last decades.^2–6^ While symptoms typically gradually subside within seven to 14 days,^7–10^ some athletes show prolonged persisting symptoms, and some never recover fully.^8,11^ When three symptoms last three months, this has commonly been defined as a post-concussion syndrome (PCS). Because of difficulties defining the syndrome,^12–15^ the descriptive term persistent post-concussive symptoms (PPCS), referring to symptoms persisting beyond the normal recovery period, is increasingly used. Vestibular dysfunction, manifested as vertigo, dizziness, unsteadiness, and visual impairment, are common in PPCS^16,17^ and, when present, associated with a worse outcome and a prolonged recovery.^18–22^

The vestibular system is an intricate sensorimotor system responsible for detection of self-motion, head and body positioning, motor responses, and multi-sensory integration with the main purposes of gaze stability and maintaining balance.^23,24^ It includes peripheral structures of the inner ear and vestibular nerves, as well as central structures including cerebellar tracts, brainstem, and supratentorial regions.^25^ The cause of persisting vestibular symptoms has not been established. While some SRC studies indicate a central origin,^26–28^ others suggest a peripheral cause,^29,30^ and recently a combination of vestibular lesions was suggested.^31^

Our aim was to establish whether vestibular dysfunction in athletes with SRC who have PPCS is of central, peripheral, or combined origin. We used morphological, diffusion tensor imaging (DTI), and diffusion kurtosis imaging (DKI) 7T magnetic resonance imaging (MRI) of the cerebellum, a detailed vestibular testing protocol, and self-reporting questionnaires addressing symptoms of post-concussion dizziness, anxiety, and depression.

## Methods

### Ethics

The study was conducted in accordance with the Declaration of Helsinki and approved by the Regional Ethics Committee, Lund, Sweden (Dnr 2017/1049). All participants received oral and written information and signed a written consent.

### Study population

Athletes ≥ 18 years old with a history of at least one SRC and experiencing symptoms exceeding six months, and healthy control athletes with no previous SRC and exercising three times per week were included. Subjects with a previous or current self-reported neurological or psychiatric disorder were not eligible for inclusion.

Athletes with SRC were recruited via rehabilitation physicians, team physicians, physiotherapists, contacts within Swedish sports societies, and via word-of-mouth. Controls were recruited by advertisement or personal contacts and were age- and sex-matched to the athletes with SRC. One researcher (AG) interviewed potential study participants and included those who fit the inclusion criteria.

### Symptom evaluation forms

#### SCAT5

The Sport Concussion Assessment Tool, 5th edition,^32^ has a graded symptom checklist evaluating 22 symptoms, where “0” is unaffected and “6” is maximum severity giving a maximum symptom severity score of 132. The questionnaire was self-administrated by the athletes with SRC.

#### DHI

The Dizziness Handicap Inventory is a self-reported questionnaire assessing the physical, functional, and emotional components of vestibular dysfunction. The total score is 0–100, where a high score indicates a high level of self-perceived handicap of vestibular dysfunction.^33^ The DHI is one of the most widely used scales for measuring the impact of dizziness on quality of life.^34^

#### HADS

The Hospital Anxiety Depression Scale is a self-reported measure of anxiety (HADS-A) and depression (HADS-D). The questionnaire has seven statements for anxiety and seven for depression, with a total score of 0–42 points. A high score implies a high level of self-perceived symptoms.^35,36^

### 7T MRI

The MRI was performed with an actively shielded 7T scanner (Achieva, Philips, Best, Netherlands) equipped with a two-channel transmit and 32 channel receive phased-array head coil (Nova Medical, Wilmington, MA). B1 inhomogeneities were reduced by using dielectric pads (Teeuwisse, Brink, & Webb, 2012). The MRI protocol consisted of three-dimensional T1-weighted imaging, DTI, and DKI. Further details are provided in Supplementary Methods 1.

#### Volumetrics

**FIG. 1.**
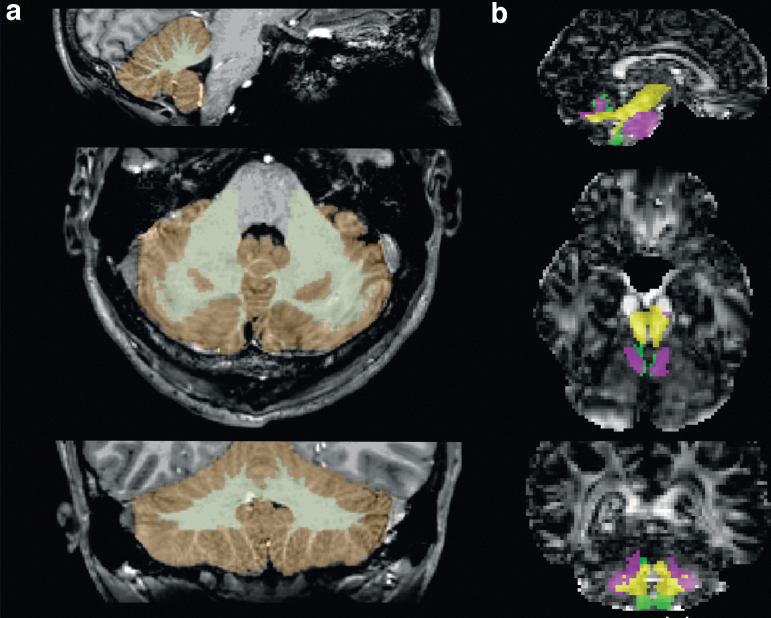
Volumetrics, diffusion tensor imaging (DTI) and diffusion kurtosis imaging (DKI) of the cerebellum. Sagittal (upper row), axial (middle row), and coronal (lower row) views of the cerebellum are shown. (a) Cerebellar segmentation. Visualization of the performance of semi-automatic FreeSurfer approach for cerebellar white matter and gray matter segmentation. White matter is depicted with yellow color and gray matter with brown color. (b) Segmentation of white matter cerebellar tracts. Visualization of the performance of TractSeg in DKI. The superior cerebellar tract is depicted in yellow, the middle cerebellar tract in purple, and the inferior cerebellar tract in green. The cerebellar tracts are depicted on top of the fractional anisotropy image. A similar segmentation of the cerebellar tracts was also performed with TractSeg in DTI.

Anatomical regions of interest were segmented from the T1w images using FreeSurfer^37^ and cerebellar white and gray volumes were analyzed (Fig. 1a). An expert reader (ENK) inspected and corrected the material for misclassification.^38^

#### DTI and DKI

The DTI and DKI processing included denoising,^39^ correction for Gibbs-ringing artefacts,^40^ brain extraction and correction of distortions from head motion and eddy currents,^41,42^ and median filtering (for DKI only). The DTI parameters were estimated through DTIFIT in FSL^43^ and DKI parameters through dipy and its module DiffusionKurtosisModel.^44^ Tract segmentation was performed using TractSeg.^45–47^ Three cerebellar tracts were selected for analysis: the inferior (left and right merged), middle, and superior (left and right merged) cerebellar tract (Fig. 1b). The fractional anisotropy (FA) and mean diffusivity (MD) were analyzed for DTI and mean kurtosis (MK), axial kurtosis (AK), and radial kurtosis (RK) for DKI.

### Vestibular tests

The vestibular tests included video head impulse test (vHIT), caloric test, cervical vestibular evoked myogenic potentials (cVEMP), videonystagmography (VNG), posturography, pursuit eye movements (PEM), and an audiogram. Before testing, the subjects were examined to exclude middle ear pathology, eventual ear wax was removed, and an audiogram was obtained. Tests were performed by two experienced audiologists according to manufacturer's instructions.

A blinded assessment by a physician specialized in neurotology (MM) was made. Tests were graded as normal or pathological whereafter an overall assessment was performed and deficits were classified as peripheral, central, or of combined origin. Peripheral signs included impairments of vHIT, caloric test, cVEMP, and a peripheral pattern of the VNG. Central signs included a central pattern of the VNG (i.e., gaze shifting nystagmus, continuous positional nystagmus), PEM, and posturography. If both peripheral and central test-deficits were found, the pathology was classified as combined. For additional details see Supplementary Methods 2.

#### vHIT

The vHIT evaluates the vestibulo-ocular reflex (VOR), which depends on the integrity of the vestibular nerve and the semicircular canals.^48^ Three vHITs were performed, one for each pair of semicircular canals.^49,50^ Evaluation was performed by calculating gains in the VOR, but mainly by classifying responses as normal or abnormal, because of the risk of possible shortcomings in calculations within the measuring system, especially for the vertical canals.^51^

#### Caloric testing

The caloric test evaluates unilateral peripheral deficits of the lateral semicircular canals and the function of the VOR. A shift in temperature of the endolymph in the semicircular canals stimulates the ampulla, causing an imbalance of the left and right VOR, resulting in nystagmus.^52–54^ Pathological outcome was defined as a reaction differing 25% or more from the opposite side.

#### cVEMP

The cVEMP is a test of the ipsilateral saccule and, hence, the inferior branch of the vestibular nerve. Central deficits delay the myogenic response, while peripheral deficits give an absent or reduced response.^55^ Evaluation was performed both by inspecting the recorded curve and by assessing calculated amplitudes, latencies, and asymmetries. The overall responses were classified as normal or abnormal, for one or both sides, according to standardized laboratory procedures.

#### Videonystagmography

The VNG registers the eye movement during different gaze directions, body positionings, and head movements. The response pattern was classified as normal, peripheral, or central. Nystagmus with a peripheral pattern beats toward the contralateral side, improves with gaze toward the lesion, worsens with gaze away from the lesion, and suppresses with visual fixation. A central pattern can be solely vertical or torsional, is not affected by fixation, and can change direction with the gaze.^56^

#### Posturography

The posturography tests integration of somatosensory, visual, and gravitational signals, and therefore studies the collective vestibular system. The recorded body sway was analyzed for frequency peaks and the variance of the forces actuated against the support surface during the body sway.^57^

#### PEM

In the PEM-test, the ability to fixate and track a visually moving object is evaluated. Visual information travels to the middle temporal area and frontal eye field of the cerebrum, to the oculomotor regions of the brainstem, projects to the striatum and cerebellum, back to the brainstem before controlling eye movement.^58^ Hence, it is a test of comprehensive visual function and depends on several central structures, including the cerebellum. Abnormal PEM was defined as prominently reduced velocity versus the stimuli, saccadic, or loss of pursuit.

### Statistics

The data were analyzed using SPSS 25 (SPSS Inc., version 25, IBM Corporation, Armonk, NY). The significance threshold was set to 0.05 for all tests but the DTI and DKI where a Bonferroni correction was made to adjust for the three cerebellar tracts, and the significance threshold was set to 0.017. The metrics test different features, why they were not corrected for multiple comparison.^59^

Time is reported as mean values with ± standard deviations (SD), or with a time-range interval. The vestibular tests were reported dichotomously. Data were analyzed for normal or skewed distribution using a Shapiro-Wilk test and histograms. Normally distributed data were compared using means and analyzed with *t* tests and analysis of variance regression analysis. Skewed or categorical data were presented as median and interquartile range (IQR) and analyzed with a Mann-Whitney *U* test for pairwise comparisons and with a chi- square test for categorical and binominal values.

## Results

### Study population

Forty-two subjects were included: 21 athletes with previous SRCs and persisting symptoms and 21 healthy age- and sex-matched controls. One patient did not complete the MRI. Demographics are listed in Table 1. Mean age was 26 (range 18–43) years, and 60% were males.

**Table 1.** Demographics

	SRC athletes,* n* = 21	Controls,* n* = 21
Male sex, % (*n*), *p* = 0.346	67% (14)	52%, (11)
Age, mean (SD), *p* = 0.404	26 (6.5) years	25 (4.2) years
Sports (*n*)	Ice hockey (7), soccer (4), karate (4), handball (2), indoor hockey (2), wrestling (1), and endurance riding (1)	Running, weightlifting, cycling, and swimming
Years of sports practice, mean (SD)	18 (5) years	-
Number of SRCs, mean (range)	5 (1–20)	-
Age at first SRC, mean (SD)	18 (5) years	-
Time from first SRC, mean (SD)	9 (7) years	-
Time from last SRC, mean (SD)	2.5 (3) years	-

Demographics of athletes with sports-related concussion (SRC) and controls.

Demographic details on the 42 participants of the present study—21 athletes with SRC and 21 athletic controls; the majority were males and of young age. The sports that the athletes with SRC were involved in are listed in declining order; because controls usually were involved in multiple athletic activities specific numbers are not listed. SD, standard deviation.

### Symptom evaluation forms

**FIG. 2.**
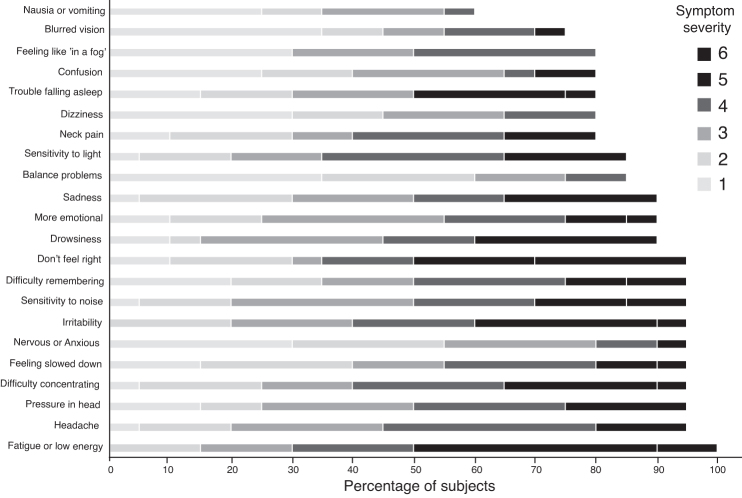
Sport Concussion Assessment Tool (SCAT). The SCAT was used for symptom grading of symptoms where the most common symptom was fatigue or low energy, reported by 100% of the athletes with sports-related concussion (SRC). Balance problems were reported by 85% and dizziness by 80% of athletes with SRC. Symptom severity was reported in seven scales where 0 is that the symptom is absent, 1 represents a mild degree of suffering from the symptom, and 6 the maximum suffering from the symptom.

On SCAT5, athletes wth SRC reported a median 20 symptoms (IQR 20–22) and symptom severity 64 (IQR 44.5–81.5). Fatigue was reported by everyone, and the least common symptoms, nausea or vomiting, by 60%. Symptoms of vestibular disturbance were reported by a vast majority (Fig. 2). Number of SRCs, age, or sex did not correlate with SCAT5 symptom severity, *p* = 0.347, *p* = 0.204, and *p* = 0.343*,* respectively.

Athletes with SRC reported higher scores on DHI compared with controls (Table 2). The number of previous SRCs, age or sex did not correlate with the DHI score, *p = 0.356*, *p = 0.147* and *p = 0.603,* respectively.

**Table 2.** Hospital Anxiety and Depression Scale and Dizziness Handicap Inventory

	SRC athletes, median (IQR)* n* = 21	Controls, median (IQR)* n* = 21	*P*
DHI	40 (27–55)	0 (0–0)	**< 0.001**
Physical	14 (9–20)	0 (0–0)	
Emotional	12 (5–19)	0 (0–0)	
Functional	14 (5–20)	0 (0–0)	
HADS	16 (11.5–19)	4 (2.5–6)	**< 0.001**
Anxiety	9 (5.5–11.5)	3 (2–4.5)	
Depression	7 (5–8.5)	1 (0–2)	

Hospital Anxiety and Depression Scale (HADS) and Dizziness Handicap Inventory (DHI). Median scores and interquartile range (IQR) for the DHI for physical, emotional, and functional subgroups and the HADS)for anxiety and depression. Total scores of the DHI and DHI differ significantly comparing athletes with sports-related concussion (SRC) with controls, *p* < 0.001 and *p* < 0.001. The significance threshold was set to 0.05; significant values are bolded.

Athletes with SRC reported elevated rating on HADS anxiety and depression subscale compared with controls (Table 2). The number of SRCs, age, or sex did not correlate with the HADS score, *p* = 0.316, *p* = 0.684, and *p* = 0.817, respectively.

### 7T MRI: volumetrics, DTI, and DKI

Because of artefacts, two athletes and three controls were excluded from the volumetric segmentations and one control from the DKI. The DTI data were missing for one athlete and DKI data from two athletes. Images were reviewed by an independent neuroradiologist and a researcher (IBB), and no structural abnormalities were observed.

**FIG. 3.**
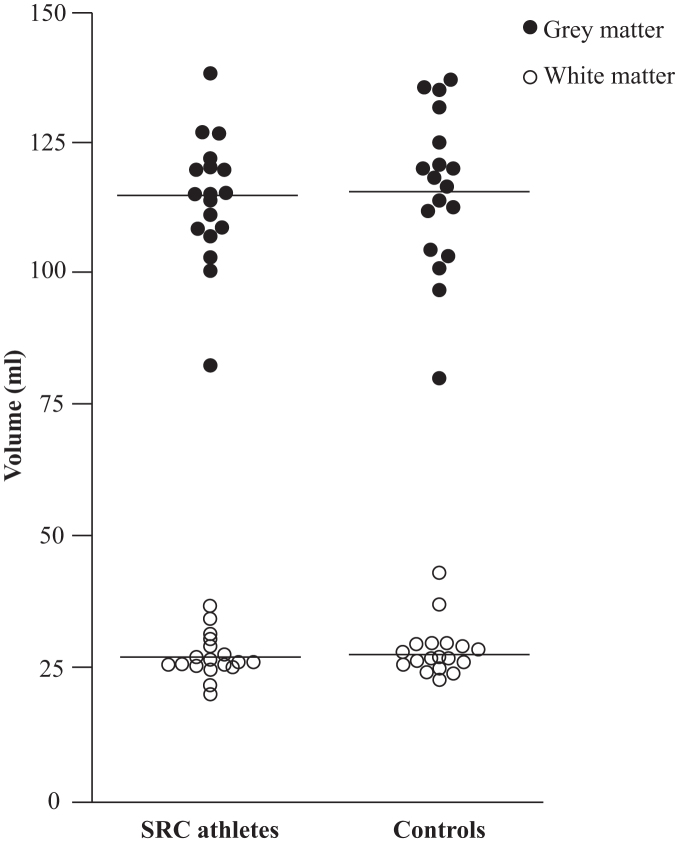
Cerebellar volumes. Cerebellar volumes of grey and white matter in 18 SRC athletes and 18 controls. Mean volumes are noted with a line. Athletes with sports-related concussion (SRC) had a white matter volume of 27.3 mL and controls of 28.5 mL (*p* = 0.441); athletes with SRC had a grey matter volume of 114 mL and controls of 116 mL (*p* = 0.722). The volumes did not differ between the groups.

Cerebellar white matter volume was 27.3 ± 4.0 mL in athletes with SRC and 28.5 ± 4.8 mL in controls; grey matter volume was 114 ± 12.2 mL in athletes and 116 ± 14.9 mL in controls, similar between groups (*p* = 0.441 and *p* = 0.722, respectively; Fig. 3), and there was no correlation with vestibular dysfunction (*p* = 0.361 and *p* = 0.774, respectively). The DKI metrics revealed a decrease in MK in the superior and inferior cerebellar tract and in RK in the superior cerebellar tract for athletes with SRC compared with controls (Table 3). No DTI or DKI metric showed significant correlation with vestibular dysfunction. TractSeg analysis found similar cerebellar tract volumes in athletes with SRC and controls using DTI and DKI (data not shown).

**Table 3.** Diffusion Weighed Imaging

DTI		SRC athletes median (IQR)* n* = 19	Controls median (IQR)* n* = 21	*P*
FA	Superior cerebellar peduncle	0.427 (0.412–0.442)	0.424 (0.397–0.448)	0.968
	Middle cerebellar peduncle	0.471 (0.448–0.488)	0.482 (0.460–0.497)	0.180
	Inferior cerebellar peduncle	0.430 (0.412–0.456)	0.433 (0.393–0.462)	0.888
MD	Superior cerebellar peduncle	0.742 (0.706–0.809)	0.740 (0.725–0.774)	0.695
	Middle cerebellar peduncle	0.745 (0.727–0.765)	0.743 (0.723–0.777)	0.715
	Inferior cerebellar peduncle	0.739 (0.686–0.829)	0.715 (0.674–0.774)	0.448

DKI		SRC athletes median (IQR)* n* = 18	Controls median (IQR)* n* = 20	*P*
MK	Superior cerebellar peduncle	0.923 (0.821–0.960)	0.972 (0.927–1.013)	**0.010**
	Middle cerebellar peduncle	0.942 (0.834–0.1.016)	0.980 (0.948–1.022)	0.090
	Inferior cerebellar peduncle	0.898 (0.860–0.953)	0.962 (0.934–0.993)	**0.010**
AK	Superior cerebellar peduncle	0.846 (0.822–0.882)	0.851 (0.829–0.891)	0.726
	Middle cerebellar peduncle	0.933 (0.906–0.955)	0.934 (0.920–0.955)	0.792
	Inferior cerebellar peduncle	0.952 (0.912–0.975)	0.945 (0.934–0.984)	0.748
RK	Superior cerebellar peduncle	0.878 (0.651–1.014)	1.032 (0.912–1.083)	**0.006**
	Middle cerebellar peduncle	0.873 (0.693–1.070)	0.999 (0.910–1.082)	0.070
	Inferior cerebellar peduncle	0.829 (0.686–0.945)	0.950 (0.851–1.023)	0.021

7T magnetic resonance imaging diffusion weighed imaging. Diffusion tensor imaging (DTI) metrics for fractional anisotropy (FA) and mean diffusion (MD), and diffusion kurtosis imaging (DKI) metrics for mean kurtosis (MK), axial kurtosis (AK), and radial kurtosis (RK), in the superior, middle, and inferior cerebellar peduncles. The significance threshold was set to 0.017 after Bonferroni correction; significant values are bolded. SRC, sports-related concussion.

### *Vestibular test* **s**

**FIG. 4.**
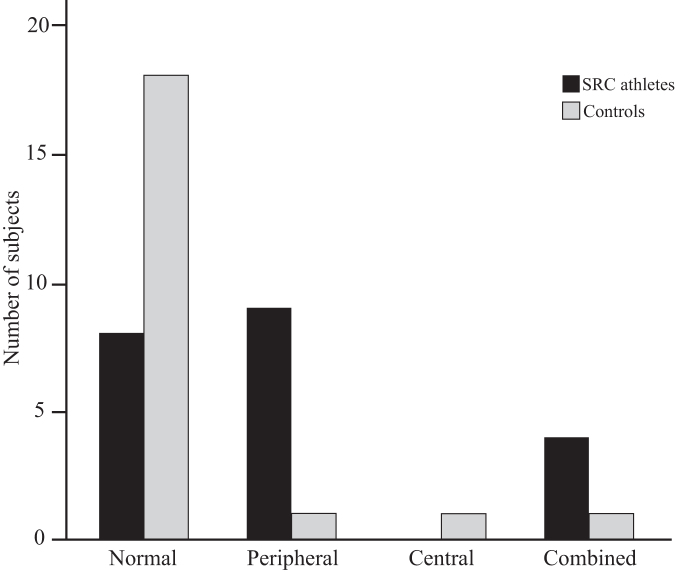
Type of vestibular pathology. Vestibular dysfunction classified as normal (non-existing), peripheral, central, or combined in athletes with sports-related concussion (SRC) and controls. Among the athletes with SRC, nine had a peripheral and four a combined deficit where none had a central pathology. Among controls, one diagnosis was peripheral, one central, and one combined pathology.

Vestibular dysfunction was present in three of 21 controls and 13 of 21 athletes with SRC (*p* = 0.001; Fig. 4). Vestibular dysfunction of peripheral (*n* = 1), central (*n* = 1), or combined (*n* = 1) origin was diagnosed in controls and of peripheral (*n* = 9) or combined (*n* = 4) origin in athletes with SRC (Fig. 4). All participants had an normal audiogram, except one athlete with SRC who had left-sided peripheral vestibular dysfunction although right-sided hearing impairment.

The athletes with SRC had worse results in vHIT and cVEMP; all test-results are listed in Table 4. The combination of pathology on the posterior semicircular canal in vHIT and the ipsilateral cVEMP argues for an injury to the inferior vestibular nerve because responses are dependent on the integrity of the posterior semicircular canal (vHIT) or the saccule (cVEMP), both innervated by the inferior vestibular nerve. Vestibular dysfunction did not correlate with the number of previous SRCs (*p =* 0.971), age (*p* = 0.141), sex (*p* = 0.758) or SCAT-5 symptom severity (*p* = 0.418).

**Table 4.** Vestibular Tests

Test name	Test structures	SRC athletes,* n* = 21 Abnormal test results		Controls,* n* = 21 Abnormal test results		*P*
		Number of subjects	Percent of subjects	Number of subjects	Percent of subjects	
vHIT	Peripheral system - Vestibular nerve, semicircular canals	**10**	**52%**	**0**	**0%**	**< 0.001**
Caloric test	Peripheral system - Semicircular canals	5	24%	4	1%	0.707
cVEMP	Peripheral system - Inferior vestibular nerve, Saccule	**8**	**38%**	**0**	**0%**	**0.002**
VNG	Central or peripheral system	3	14%	0	0%	0.072
Posturography	Collective vestibular system	8	38%	3	14%	0.079
PEM	Central system	4	19%	2	10%	0.378

Vestibular tests.

The results of vestibular tests for athletes with sports-related concussion (SRC) and controls. The results for the specific tests video head impulse test (vHIT), caloric test, cervical evoked myogenic potentials (cVEMP), videonystagmography (VNG), posturography, and pursuit eye movements (PEM) are reported as number and percent with pathological findings. The results of vHIT and cVEMP differ significantly between athletes with SRC and controls. The significance threshold was set to 0.05; significant values are bolded.

**FIG. 5.**
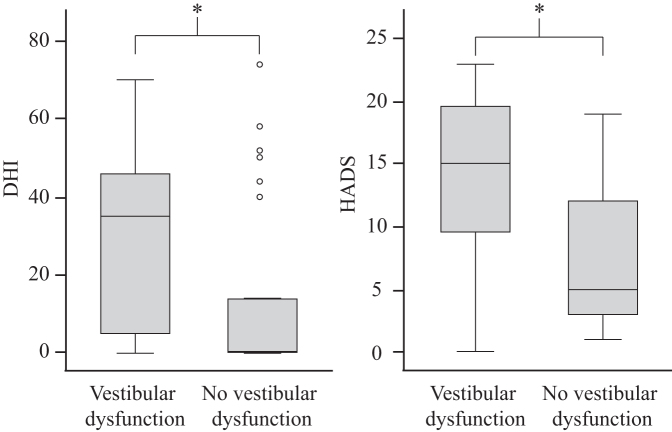
Dizziness Handicap Inventory (DHI) and Hospital Anxiety and Depression Scale (HADS). Total scores of the DHI and HADS in athletes with and without vestibular dysfunction. If a vestibular pathology existed, subjects scored higher on DHI (*p* = 0.019) and HADS (*p* = 0.004) than athletes without vestibular dysfunction. *p < 0.05.

Subjects with vestibular dysfunction assessed higher scores on DHI (median 35, IQR 4.5–47 vs. median 0, IQR 0–20.5, *p* = 0.019) and HADS (median 15, IQR 9.25–19.75 vs. median 5, IQR 3–12.25, *p* = 0.004) compared with subjects without vestibular dysfunction (Fig. 5).

## Discussion

In this study, our focus was to investigate the vestibular system of athletes with persisting symptoms for ≥6 months after SRC. Athletes with SRC had a high burden of self-perceived symptoms and a negative impact on health because of dizziness, depression, and anxiety. The main finding was that athletes with SRC had a peripheral vestibular deficit, specifically dysfunction of the inferior vestibular nerve, whereas there was no difference in cerebellar gray and white matter as evaluated by 7T MRI.

In the present study, 80% and 85% of athletes with SRC reported dizziness and imbalance, respectively, which are symptoms of vestibular dysfunction. The included athletes with SRC had a high level of self-perceived symptoms on SCAT, DHI, and HADS, and subjects with vestibular dysfunction had higher levels of symptoms on DHI and HADS. These findings are in line with previous studies showing that vestibular dysfunction negatively affects SRC outcome.^18–22^ The DHI was developed to measure self-perceived handicap from symptoms of vestibular dysfunction and how they impact quality of life (QoL).^33,34^ After SRC, athletes commonly have increased levels of anxiety and depression,^60^ and PPCS can lead to a lower QoL.^61^

White matter pathology is considered a major contributor to the sequelae of SRC,^62^ and a central cause of the vestibular dysfunction has been suggested. Two previous studies observed no abnormality in functional tests of the peripheral vestibular structures, including cVEMP and vHIT, at 10–14 days post-SRC. While the central vestibular systems were not tested, a central cause was suggested.^27,28^ Similarly, peripheral vestibular deficiency was not found in children and adults 4–6 months post-concussion.^63^

Although not observed on conventional neuroimaging, the rotational forces sustained at impact are believed to disrupt white matter integrity.^64,65^ The DTI is sensitive to the microstructural integrity of white matter fibers and axonal injury.^66–68^ In SRC, DTI has revealed reduced white matter integrity, not explained by structural damage or volume loss.^64,69–71^ In patients with mTBI examined 22 days post-injury, a decreased cerebellar FA and increased MD correlated with vestibular dysfunction.^26^ Vestibular laboratory findings were not reported, and the patients with mTBI differed in many aspects from the athletes with SRC in our present study.

To date, only one previous study investigated both the peripheral and central vestibular systems, examining a cohort of patients with mixed-TBI who had imbalance or dizziness 2–77 days post-injury.^31^ A decreased FA and increased MD was observed, correlating with vestibular dysfunction.^31^ These results are in contrast to our present 7T DTI and volumetry findings, although these studies differ concerning selected patients, time points, mechanism, and injury severity. Decreased MK and/or RK in the DKI metrics were apparent in two cerebellar tracts, not confirmed by DTI, volumetrics, or by central vestibular tests. The high signal-to-noise ratio (SNR) of 7T MRI scanners allows for high-resolution structural imaging in feasible scan times, and the high image contrast can aid detection of diffuse pathology.^72^

Arguably, these subtle findings on 7T MRI suggest discrete white matter changes insufficient to explain the impairment of the vestibular tests. If central white matter changes were more substantial, widespread alterations in the cerebellar tracts on both DKI and DTI would be expected and confirmed with a central pattern on vestibular laboratory tests. The subtle DKI findings could explain why some athletes with SRC presented with a combined deficit on vestibular tests. Further, this study does not address supratentorial white matter alterations, which may also cause vestibular symptoms if pronounced.

Some previous reports have suggested a peripheral origin of vestibular dysfunction. A retrospective study found that 26% of children and adolescents with vestibular disturbance had a peripheral vestibular disorder four months post-SRC.^29^ Not all patients underwent vestibular testing, however.^29^ In patients with TBI who had vestibular symptoms, acute unilateral peripheral vestibular loss was found in 19%,^30^ and postmortem studies revealed degeneration of the superior and inferior vestibular nerves.^73,74^

We observed that many athletes with SRC differed from controls on vHIT, cVEMP, VNG, and posturography, with a specific pattern of dysfunction suggesting a peripheral vestibular pathology, in contrast to some earlier reports.^27,28,63^ Athletes with SRC had abnormal results in the posterior semicircular canal in vHIT and the ipsilateral side in cVEMP. The inferior branch of the vestibular nerve is tested directly by cVEMP and through the posterior semicircular canal and sacculus by vHIT. Both vHIT and cVEMP confirmed the location of injury to the inferior vestibular nerve, in a repeatable manner. While the caloric test, VNG, PEM, and posturography were inconclusive, they do not exclude an inferior vestibular nerve injury.

On 7T MRI, no difference in cerebellar volumes or DTI metrics was observed and only minor changes in DKI metrics, arguing against central structural pathology and supporting the hypothesis of a peripheral vestibular injury.

The mechanisms causing a lesion of the inferior posterior nerve have not been established. In vestibular neuritis, the superior branch of the vestibular nerve is more affected than the inferior one,^75,76^ attributed to the longer route through the temporal bone.^77^ Reversely, a longer bony canal may protect the nerve better in case of acceleration-deacceleration relative to the skull base, resulting in lesions to the inferior nerve. Although hypothetical, this might be a plausible pathophysiological mechanism for our findings.

### Limitations

We evaluated a highly characterized cohort of athletes with SRC who had persistent post-concussive symptoms ≥6 months. Thus, our cohort may not be representative of all athletes with SRC. Because we had no control group of recovered athletes with SRC nor athletes evaluated at an earlier post-injury time, other results could have been achieved in such cohorts. The selected groups and relatively small sample size may compromise generalizability and correlation analyses.

Although 7T MRI enables the acquisition of T1w images at a submillimeter resolution, subcortical image artefacts caused by B1+ inhomogeneity^78^ appeared in some subjects. The motivation for using 7T MRI is its higher SNR compared with 3T MRI.^79^ The 7T MRI, however, suffers from greater susceptibility-related artefacts,^80,81^ which explains the artifactual foreshortening of the brainstem in the anteroposterior direction seen in Fig. 1b. Moreover, the shorter T2 relaxation times compared with 3T partially cancels the relative SNR-benefit of 7T, especially for diffusion MRI, which necessitates longer echo times.^82^ Combing ultra-high field strengths with ultra-strong gradients could mitigate that problem.^83,84^ Slightly higher effect sizes, however, have been found at 7T compared to 3T.^85^.

The size of a tract is known to affect the performance of TractSeg, but merely in small tracts such as the fornix and the anterior commissure. The cerebellar tracts, however, are sufficiently large and should not be affected.^46^

Vestibular laboratory tests are sensitive and can be influenced by tiredness and previous MRI.^86^ To avoid these sources of error, the athletes with SRC and controls did their examinations well-rested during the midmorning and before the MRI. Several of the vestibular tests are challenging to interpret, because the results may both indicate a peripheral or central deficit, may be influenced by side differences, or body positioning and movement during the examinations. Therefore, we chose not to compare the specific numbers between the groups but instead compare pathological from normal responses. This classification was done by a neurotology specialist (MM), who was blinded to injury status, MRI findings, and questionnaire results.

### Future use of 7T MRI

Ultrahigh field 7T diffusion MRI has the potentials to characterize human morphology and neuronal substructure, providing exquisite anatomical details and delineation of fiber substructures. Findings in substructures have been reported in the cochlear nucleus,^87^ pedunculopontine nucleus, and surrounding white matter tracts^82^ and brainstem tracts.^88^ Moreover, promising methods have been developed for improving image quality in 7T diffusion weighed MRI, which could subsequently increase the effectiveness of ultrahigh field diffusion MRI,^79,89^ summarized in the article by Gallichan.^90^ Interestingly, one direction could be the fusion of 3T and 7T, exploiting the perks of both worlds, with high angular and spatial resolution, respectively.^91^

## Conclusion

An SRC may induce a multitude of pathologies in the peripheral and/or central parts of the vestibular system. Establishing an etiology of the impairment is crucial because it may guide intervention and clinical management. Our study, investigating both central and peripheral dysfunction, strongly implies a peripheral nerve dysfunction associated with vestibular dysfunction in athletes with SRC who have persistent post-concussive symptoms.

## Supplementary Material

Supplemental data

Supplemental data

## Supplementary Material

Supplementary Method 1

Supplementary Method 2
