# Supplementary material for: Post-Concussive Vestibular Dysfunction Is Related to Injury to the Inferior Vestibular Nerve
Source: J Neurotrauma. 2022 Jun 3;39(11-12):829–40. doi: 10.1089/neu.2021.0447 (PMC9225415; doi:10.1089/neu.2021.0447)
Supplement: Supplemental data [file Supp_Method1.docx]

**Supplementary Methods 1.** MRI

The MRI protocol consisted of (a) 3D T1-weighted images (FOV: 230x230x180 mm^3^, resolution 0.80x0.80x0.80 mm^3^, TR/TE: 8.00/1.97 ms); (b) DTI (FOV: 224x224x110 mm^3^, resolution 2x2x2 mm^3^, TR/TE: 9200/65 ms, two b = 0 s/mm^2^ volumes with opposing polarities of the phase-encode blips. Diffusion encoding was applied in six directions with b = 100 s/mm^2^ and 30 directions with a b-value of 1000 s/mm^2^); (c) DKI (FOV: 224x224x120 mm^3^, resolution 2x2x2 mm^3^, TR/TE: 9800/76 ms, two b = 0 s/mm^2^ volumes with opposing polarities of the phase-encode blips, six b = 100 s/mm^2^ volumes, six b = 500 s/mm^2^ volumes, 10 b = 1000 s/mm^2^ volumes and 30 volumes with a b-value of 2000 s/mm^2^).

For volumetric segmentation, an expert reader (ENK) inspected each three-dimensional segmentation image for points of misclassification of white matter, grey matter, and pial surface boundaries in two separate editing steps^1^. Following initial labeling, images were inspected for white matter omissions and control points were added, extending the boundaries of white matter segmentation to accurately incorporate white matter tracts. Subsequently, the images were inspected for erroneous classification of white matter regions to grey matter or vice versa and edits were made to the parcellation image to redraw white matter and grey matter boundaries. All images were double-checked by two trained supervisors (MN and NM) to ensure that edits were implemented correctly.

DTI processing comprised four steps: denoising, correction for Gibbs-ringing artefacts, brain extraction and correction of distortions due to head motion and eddy currents. Denoising was performed using Marchenko-Pastur principal component analysis^2^. For mitigating Gibbs-ringing artefacts we used the method proposed by Kellner et al^3^. Motion and eddy currents correction was applied using the eddy method provided by FSL^4^. Geometric distortions were corrected using topup by FSL^5^. DKI processing comprised of those same four steps plus the application of median filtering as a fifth step and prior to the estimation of the diffusion parameters. DTI parameters were then estimated through DTIFIT in FSL using weighted linear least squares^6^. DKI parameters were computed using the package dipy and its module DiffusionKurtosisModel, with weighted linear least squares being the fitting to the model method too^7^. To obtain tract-specific diffusion parameter values, white matter tract segmentation was performed using TractSeg^8-10^, that automatically segments 72 major white matter tracts.

**References**

1. Ross, MC., Dvorak, D., Sartin-Tarm, A., Botsford, C., Cogswell, I., Hoffstetter, A., Putnam, O., Schomaker, C., Smith, P., Stalsberg, A., Wang, Y., Xiong, M., and Cisler, JM. (2021). Gray matter volume correlates of adolescent posttraumatic stress disorder: A comparison of manual intervention and automated segmentation in FreeSurfer. Psychiatry Res Neuroimaging 313, 111297.

2. Veraart, J., Novikov, DS., Christiaens, D., Ades-Aron, B., Sijbers, J., and Fieremans, E. (2016). Denoising of diffusion MRI using random matrix theory. Neuroimage 142, 394-406.

3. Kellner, E., Dhital, B., Kiselev, VG., and Reisert, M. (2016). Gibbs-ringing artifact removal based on local subvoxel-shifts. Magn Reson Med 76, 1574-81.

4. Anderson, EC., and Thompson, EA. (2002). A model-based method for identifying species hybrids using multilocus genetic data. Genetics 160, 1217-29.

5. Andersson, JLR., and Sotiropoulos, SN. (2016). An integrated approach to correction for off-resonance effects and subject movement in diffusion MR imaging. Neuroimage 125, 1063-78.

6. Basser, PJ., Mattiello, J., and LeBihan, D. (1994). MR diffusion tensor spectroscopy and imaging. Biophys J 66, 259-67.

7. Jensen, JH., Helpern, JA., Ramani, A., Lu, H., and Kaczynski, K. (2005). Diffusional kurtosis imaging: the quantification of non-gaussian water diffusion by means of magnetic resonance imaging. Magn Reson Med 53, 1432-40.

8. Wasserthal, J., Neher, PF., Hirjak, D., and Maier-Hein, KH. (2019). Combined tract segmentation and orientation mapping for bundle-specific tractography. Med Image Anal 58, 101559.

9. Wasserthal, J., Neher, P., and Maier-Hein, KH. (2018). TractSeg - Fast and accurate white matter tract segmentation. Neuroimage 183, 239-53.

10. Sotiropoulos, SN., Jbabdi, S., Xu, J., Andersson, JL., Moeller, S., Auerbach, EJ., Glasser, MF., Hernandez, M., Sapiro, G., Jenkinson, M., Feinberg, DA., Yacoub, E., Lenglet, C., Van Essen, DC., Ugurbil, K., Behrens, TE., and Consortium, WU-MH. (2013). Advances in diffusion MRI acquisition and processing in the Human Connectome Project. Neuroimage 80, 125-43.
