# Supplementary material for: Post-Concussive Vestibular Dysfunction Is Related to Injury to the Inferior Vestibular Nerve
Source: J Neurotrauma. 2022 Jun 3;39(11-12):829–40. doi: 10.1089/neu.2021.0447 (PMC9225415; doi:10.1089/neu.2021.0447)
Supplement: Supplemental data [file Supp_Method2.docx]

**Supplementary Methods 2.** Vestibular tests

***vHIT***

The vHIT was performed according to manufacturer’s instructions (EyeSeeCam, Interacoustics, Middelfart, Denmark). The subject was sitting upright wearing goggles with an infrared camera that analyzes eye movement, and a motion sensor that records head movement. While the gaze was focused, head movements of 10-20 degrees for 150-200ms was induced in the plane of each pair of semicircular canals^1, 2^.

***Caloric testing***

A shift in temperature of the endolymph in the semicircular canals creates a current which stimulates the sensory components in the ampulla, causing an imbalance of the left and right VOR, resulting in nystagmus. When applying a cold temperature a fast-beating nystagmus with an opposite direction appears on the side being stimulated, and a slow-beating nystagmus on the other eye, and when applying warm temperature an opposite reaction is normally observed^3-5^.

Hot and cold (44°C and 30°C) water was rinsed into the ear and nystagmus were recorded (VisualEyes 525, Interacoustics, Middelfart, Denmark).

***cVEMP***

The cVEMP is a myogenic response of the sternocleidomastoid muscle, evoked by sound. This is primarily a test of the ipsilateral saccule and hence, the inferior branch of the vestibular nerve. Central deficits delays the response, while peripheral deficits gives an absent or reduced amplitude response^6^.

Tests were performed according to the manufacturer’s instructions (Eclipse, Interacoustics, Middelfart, Denmark). The subject was presented with 500 Hz tone burst in one ear while having the head turned to the side. Surface electrodes was placed on the contralateral sternocleidomastoid muscle and the myogenic responses recorded.

***Videonystagmography***

The subjects wore goggles with infrared cameras registering eye movements (VisualEyes 525, Interacoustics, Middelfart, Denmark). The subjects were instructed to sit upright, look to the left, right, up, down and straight ahead to register spontaneous nystagmus in different directions. To register horizontal and vertical nystagmus the subject laid on each side, and flat on the back with head flexed 15 degrees. A headshake test was done by shaking the head from one side to the other, 1-2 times per second, >10-15 seconds, while the subjects kept a central gaze position.

***Posturography***

The participant stood on a platform equipped with strain-gauge sensors looking at a focus point 1.5 m ahead or blindfolded, with and without stimulations with vibratory perturbation from the platform. The test was performed in 4 sessions, standing with open or closed eyes for 30s and with open or closed eyes for 230s followed by a 30s recording. The subjects were exposed to a pseudorandomized binary sequence of perturbations caused by a vibration towards both calf muscle. The recorded body sway were analyzed for frequency peaks and the variance of the forces actuated against the support surface during of the body sway, as described elsewere^7^.

***Pursuit eye movements***

The subjects were instructed to look at a focus point presented on a screen in front of them, the focus point moved in different directions with increasing velocity, while eye movement was recorded (VisualEyes 525, Interacoustics, Middelfart, Denmark).

**References**

1. Ulmer, E., Bernard-Demanze, L., and Lacour, M. (2011). Statistical study of normal canal deficit variation range. Measurement using the Head Impulse Test video system. Eur Ann Otorhinolaryngol Head Neck Dis 128, 278-82.

2. McGarvie, LA., Martinez-Lopez, M., Burgess, AM., MacDougall, HG., and Curthoys, IS. (2015). Horizontal Eye Position Affects Measured Vertical VOR Gain on the Video Head Impulse Test. Front Neurol 6, 58.

3. Shepard, NT., and Jacobson, GP. (2016). The caloric irrigation test. Handb Clin Neurol 137, 119-31.

4. Murphy, KA., and Anilkumar, AC. Caloric Testing. StatPearls. Treasure Island (FL)2021.

5. Bárány, R. (1906). Untersuchungen ueber den vom Vestibularapparat des Ohres reflektorisch ausgeloesten rhythmischen Nystagmus und seine Begleiterscheinungen. Monatschrift Ohrenheilkunde 193-297.

6. Rosengren, SM., Welgampola, MS., and Colebatch, JG. (2010). Vestibular evoked myogenic potentials: past, present and future. Clin Neurophysiol 121, 636-51.

7. Johansson, R., Magnusson, M., Fransson, PA., and Karlberg, M. (2001). Multi-stimulus multi-response posturography. Math Biosci 174, 41-59.
